# Clozapine induced oesophagitis: A case report

**DOI:** 10.1192/j.eurpsy.2022.874

**Published:** 2022-09-01

**Authors:** N. Herch, R. Lansari, A. Larnaout, D. Achref, W. Melki

**Affiliations:** 1 Razi Psychiatric Hospital, Psychiatry D, Manouba, Tunisia; 2 Razi Hospital, Psychiatry D, Manouba, Tunisia; 3 Razi Hospital, Psychiatry D, Beja, Tunisia

**Keywords:** side effect, clozapine, schizophrénia, oesophagitis

## Abstract

**Introduction:**

There are several case reports describing clozapine side effects such as agranulocytosis, constipation,tachycardia but rarely cases describing oesophagitis caused by clozapine were reported.

**Objectives:**

To report the first case in our country about clozapine induced oesophagitis.

**Methods:**

We describe a case in which a patient who has no gastrointestinal past history,has developed an oesophagitis stage 2 of Savary and Miller Classification without any gastroesophageal reflux disease, few weeks after introducing clozapine at therapeutic dose.

**Results:**

A 25 years old male patient with resistant schizophrenia managed with clozapine,was admitted to reinitiate his treatment after weeks of stopping his medication.During hospitalization, our patient developed a sudden haematemesis in 10 days after commencement of clozapine. The patient had no history of gastrointestinal symptoms or disease. The clinical examination and blood tests did not find any signs of bleeding severity.A gastroscopy was performed, revealing esophagitis stage 2 of Savary and Miller classification and a cardiac polyp removed with biopsy forceps that showed no malignant lesions .The patient was treated with acid suppressant therapy.There was no further episode of haematemesis and our patient healed uneventfully within a week.As for clozapine, it wasn’t interrupted and we continued increasing doses very carefully with no further incident.

**Conclusions:**

Although it is a rare side effect, oesophagitis may appear at therapeutic doses of clozapine, and this possibility should be taken into account when treating patients with resistant psychiatric disorders.

**Disclosure:**

No significant relationships.

